# A dynamic transcriptomic atlas of cytokine-induced killer cells

**DOI:** 10.1074/jbc.RA118.003280

**Published:** 2018-10-17

**Authors:** Mingyao Meng, Lin Li, Ruhong Li, Wenju Wang, Yang Chen, Yanhua Xie, Rui Han, Kai Zhu, Wenwen Huang, Lili Yang, Shuo Li, Jianlin Shi, Weiwei Tan, Hui Gao, Yiyi Zhao, Li Yang, Jing Tan, Zongliu Hou

**Affiliations:** From the ‡ Yan'an Affiliated Hospital of Kunming Medical University, Kunming 650051, Yunnan, China,; the § Key Laboratory of Tumor Immunological Prevention and Treatment of Yunnan Province, Kunming 650051, Yunnan, China, and; the ¶ Ministry of Education (MOE) Key Laboratory of Bioinformatics, Bioinformatics Division and Center for Synthetic and Systems Biology, BNRist, Department of Automation, Tsinghua University, Beijing 100084, China

**Keywords:** immunotherapy, tumor therapy, bioinformatics, immunology, computational biology, cytokine-induced killer cells, deep sequencing, cytotoxicity, WGCNA, safety, cytokine, CD8, anticancer treatment

## Abstract

Several clinical immunotherapy trials with cytokine-induced killer (CIK) cells have been reported. However, molecular evidence of cell expansion, acquisition of tumor cytotoxicity, and safety of CIK cells is required before putting them to clinical use. Here, we performed dynamic transcriptomic analyses of CIKs generated from primary peripheral blood mononuclear cells exposed to interferon-γ, OKT3, and interleukin-2. CIK mRNAs were extracted and sequenced at days 0, 1, 7, and 14 and subjected to bioinformatics analyses. Using weighted correlation network analysis (WGCNA), we identified two major gene modules that mediate immune cell activation and mitosis. We found that activation and cytotoxicity of CIK cells likely rely on cluster of differentiation 8 (CD8) and its protein partner LCK proto-oncogene, Src family tyrosine kinase (LCK). A time-course series analysis revealed that CIK cells have relatively low immunogenicity because of decreased expression of some self-antigens. Importantly, we identified several crucial activating receptors and auxiliary adhesion receptors expressed on CIK cells that may function as tumor sensors. Interestingly, cytotoxicity-associated genes, including those encoding PRF1, GZMB, FASL, and several cytokines, were up-regulated in mature CIK cells. Most immune-checkpoint molecules and inflammatory tumor-promoting factors were down-regulated in the CIK cells, suggesting efficacy and safety in future clinical trials. Notably, insulin-like growth factor 1 (IGF-1) was highly expressed in CIK cells and may promote cytotoxicity, although it also could facilitate tumorigenesis. The transcriptomic atlas of CIK cells presented here may inform efforts to improve CIK-associated tumor cytotoxicity and safety in clinical trials.

## Introduction

Adoptive immunotherapies have become one of the most important approaches to treat cancer effectively. Recent progress has proven that adoptive transfer of educated immune cells can yield strong and durable antitumor response with minimal adverse events (1). In recent decades, a variety of immune effector cells have been developed, including lymphokine-activated killer cells, cytokine-induced killer (CIK)^4^ cells, tumor-infiltrating lymphocytes (TILs), natural killer (NK) cells, γδ T cells, cytotoxic T lymphocyte (CTL) cells, and chimeric antigen receptor T cells (2–8). Lymphokine-activated killer cells were described early on to be partially effective in both animal models and patients with metastatic cancer, although severe fluid retention was a major side effect (2). TILs are prepared from infiltrated lymphocytes of resected tumor specimen and expended in the presence of IL-2. Multiple groups have reported that more than 38% of patients with melanoma who receive TILs therapy show objective response (9, 10). CTLs are *ex vivo*–generated and tumor antigen–specific CD4^+^ and CD8^+^ T cells. They have been shown to be effective in nasopharyngeal and ovarian carcinomas (11, 12). γδ T cells are T cells with a distinctive T-cell receptor that show promise in immunotherapy of renal carcinoma, melanoma, lung cancer, and leukemia (13). NK cells are innate immune lymphocytes characterized by the expression of CD56 and absence of CD3. A great number of clinical trials based on NK cells were performed in both solid and hematologic malignancies (5).

CIK cells are a heterogeneous cell population with antitumor activity against a broad array of tumor targets, including acute myeloid leukemia, B lymphoma cells, and some solid tumors (14). Several phase I trials have been performed to test clinical efficacies of CIK cells among a small number of patients (15).

Notably, the first CIK clinical trial was performed among 10 patients with metastatic renal cell carcinoma, colorectal cancer, and lymphoma (16). Complete response was observed in one patient with follicular lymphoma. A recent study reported that DC-CIK increased the 3-year disease-free survival of patients with renal cell carcinoma (17). Importantly, CIK cell treatment can be given as an adjuvant immunotherapy in combination with chemotherapy, radiotherapy, surgery, and cytokine therapy, which are effective in the treatment of renal cell carcinoma, nonsmall lung cancer, gastrointestinal cancer, and hepatocellular carcinoma (14).

CIK cells are a mixture of T lymphocytes, which comprise CD3^+^CD56^+^ and CD3^+^CD56^−^ cytotoxic T cells. Peripheral blood lymphocytes are primed with interferon-γ, which provides contact-dependent (CD58/LFA-3) and soluble (IL-12) crucial signals to promote generation of autophagy and antigen cross-presentation (18). IL-2 and OKT3 are essential for T-cell proliferation, survival, and acquisition of cytolytic function. By 14 days of cell expansion, a heterogeneous cell population was obtained with potent and MHC-unrestricted tumor cytotoxicity. Naturally, the proliferation capacity and tumor cytotoxic function of CIK cells are two major determinants of their therapeutic efficacies. Although immunotherapy based on CIK cells has been widely proven to be effective in clinical trials, the molecular evidence of cell expansion, acquisition of tumor cytotoxicity, and safety is not fully understood.

In this study, we performed dynamic transcriptomic analyses to monitor the gene expression changes during CIK cell production. The mRNAs were sampled on days 0, 1, 7, and 14 and sequenced by an ion proton sequencer. RNA-Seq data were mined by a series of advanced bioinformatic and computational analyses. Our findings should enable better understanding of the molecular mechanism of tumor cytotoxicity and the safety of CIK cell–based immunotherapy.

## Results

### Preparation of CIK cells and overview of RNA-Seq data

CIK cells were generated from peripheral blood mononuclear cells (PBMCs) of four healthy volunteers under the same conditions. PBMCs were marked as day 0 and sampled for flow cytometry and RNA extraction. Then PBMCs were primed with interferon-γ for 24 h and stimulated with OKT3 and IL-2 for 13 days. The cells were sampled at days 1, 7, and 14 for RNA extraction (Fig. S1*A*). The proliferation capacity and phenotype dynamic were monitored during CIK cell production. Absolute cell counting showed that CIK cells increase over 10-fold after 14 days of expansion (Fig. S1*B*). The average percentage of CD3^+^CD56^+^ CIK cells was over 35%, and that of CD3^+^CD8^+^ cells was over 70% (Fig. S1*C*). The total RNA-Seq raw reads of all cell samples ranged from 12.7 to 22.5 million, and more than 95% of the reads had a quality score of ≥Q20. There were approximately 13.21 ± 2.05 clean reads (90.91% of the total reads) uniquely mapped to the human genome among 16 independent samples. In the uniquely mapped reads, more than 80% of reads were aligned at exons, and the remaining were aligned at the UTR region, transcription end site, transcription start site, introns, and intergenic regions. Then we performed transcript assembly, reads per kilobases per million reads (RPKM) value normalization, and annotation. The relative expressions of the genes in CIK cell samples are shown in Table S1. To visualize the process of CIK cell induction, we performed three-dimensional principal component analysis to evaluate global gene expression variance. It showed that samples from day 0 and day 1 were closely associated, and samples from day 7 and day 14 were related (Fig. S2*A*). Significant gene expression variances were observed between cells from days 7 and 14 based on gene expression clustering (Fig. S2*B*). To characterize the functional consequences of CIK cell induction, we screened the differentially expressed genes (DGEs) between PBMCs and CIK cells by the following criteria: log_2_FC > 1 or log_2_FC < −1, false discovery rate < 0.05, and *p* < 0.05. We identified 7740 DEGs between PBMCs and CIK cells. Of these DEGs, there were 2903 and 4837 genes up-regulated and down-regulated, respectively.

### Weighted correlation network analysis (WGCNA) identifies gene clusters of cell proliferation and immune cell activation

**Figure 1.**
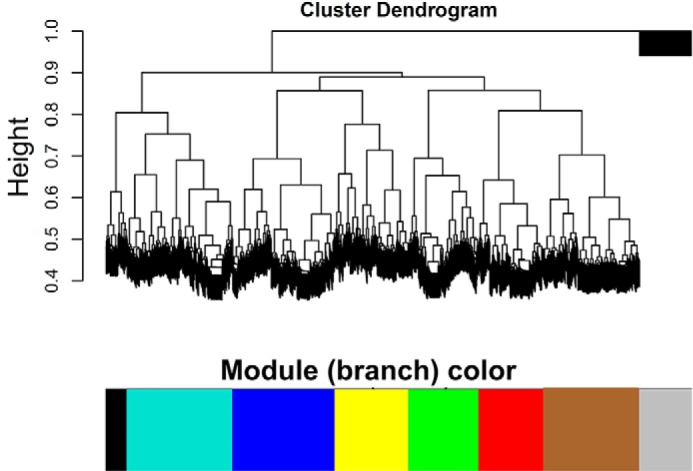
**WGCNA analysis identified modules of DGEs.** A hierarchical cluster analysis dendrogram was used to detect coexpression clusters along with seven color assignments.

**Figure 2.**
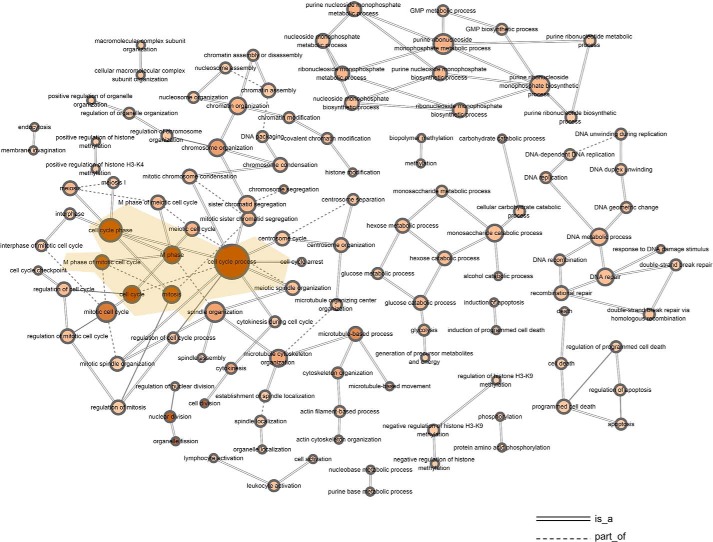
**GO tree analysis of the brown gene module, indicating correlations among GO terms.**

**Figure 3.**
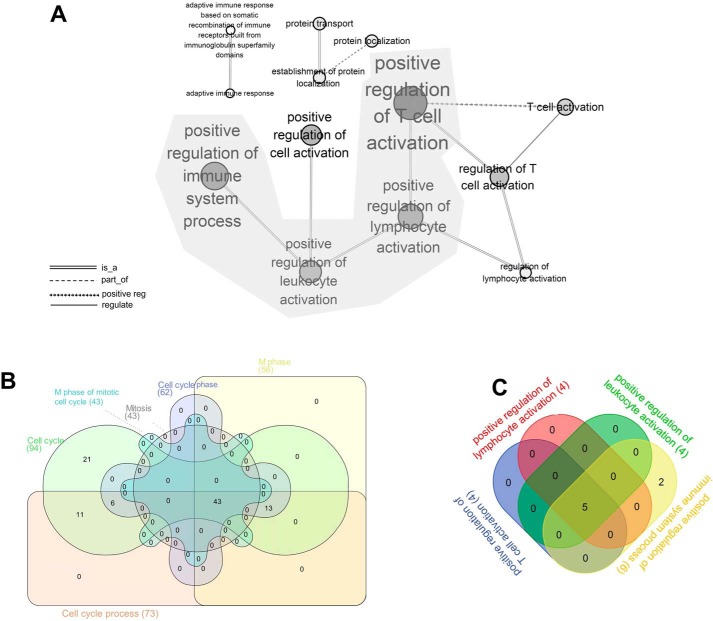
**GO tree analysis of the black gene module and Venn analysis of the essential GO terms.** *A*, GO tree analysis of the black module showed correlations among GO terms associated with immune cell activation. *B* and *C*, Venn analysis of the essential GO terms that were associated with cell cycle and T-cell activation. (The *degree* of the *color* indicates the significance of each GO term, and the *size* of the *node* shows the interactions with the surrounding nodes.)

**Figure 4.**
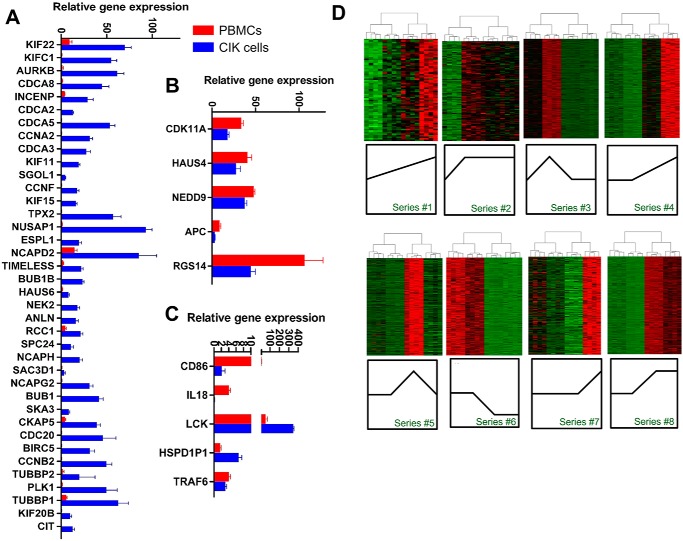
**Differential expression of crucial genes in the brown and black modules and selected temporary expression pattern of DGEs.** *A* and *B*, the relative expression of up-regulated and down-regulated genes in the *brown module* that were essential to CIK cell proliferation, respectively. *C*, the relative expression of DGEs in the *black module* that promoted immune cell activation. *D*, heat map showing the gene expression pattern at each time point for genes in that cluster. *Error bars*, S.E.

To obtain gene sets that were closely related with CIK functions, we performed WGCNA to find clusters in which genes were highly correlated. The results showed that seven modules were formulated in which DGEs were highly interconnected, and the gene modules were *colored* (Fig. 1). The genes that showed low connectivity weight were classified into a gray module. By gene ontology analyses, we found that gene sets clustered in black and brown modules were highly involved in T-cell activation and the cell cycle. The gene sets in the other five modules were involved in functions including cell death, regulation of glucose import, and regulation of transcription factor activity. Next, we included all of the GO terms of the brown module and built the GO tree based on the relations among them. The degrees of size and color were used to illustrate the interconnectedness and significance of each node. The GO tree of the brown module indicated that GO terms including cell cycle process, M phase, mitosis, cell cycle, and M phase of mitotic cell cycle were grouped and showed the most significance among all of the terms (Fig. 2). Likewise, the GO tree of the black module indicated that positive regulation of T-cell activation, lymphocyte activation, leukocyte activation, and immune system process were the most significant GO terms and strongly correlated (Fig. 3*A*). Next, Venn analysis was employed to explore the DGEs that were crucial for CIK cell differentiation. The results showed that there were 43 and 5 DGEs shared by the core GO terms in brown and black modules, respectively (Fig. 3, *B* and *C*). There were 38 genes up-regulated and 5 genes down-regulated shared by GO terms related with cell proliferation (Fig. 4, *A* and *B*). Among five DGEs in T-cell activation, *CD86*, *IL-18*, and *TRAF6* were down-regulated, and *LCK* and *HSPD1P1* were up-regulated in CIK cells (Fig. 4*C*).

### Functional analysis of dynamic gene expression profiles

**Figure 5.**
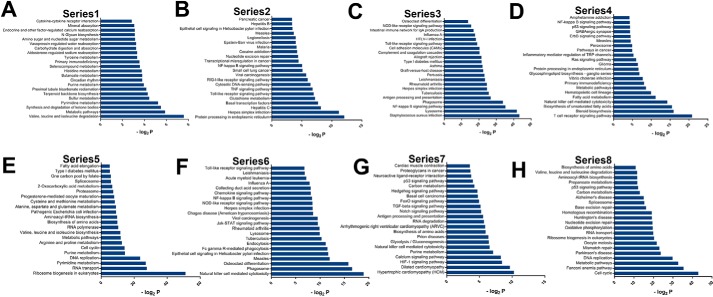
**KEGG analysis of selected series clusters of genes identified different key biological events during CIK cell production.**

To investigate stage-specific gene function during CIK cell differentiation, time series test of cluster analysis and hierarchical clustering were performed. The principle of the time series test is to classify the genes with a highly similar expression trend. There were 25 time series clusters based on the RPKM values of all genes. To further focus on the series clusters that are associated with functions of CIK cells, we performed pathway analysis based on the KEGG database. We selected eight clusters from 25 clusters ranging from PBMCs to CIK cells that were closely related with immunity and cell proliferation (Fig. 4*D*). The gene set that was persistently up-regulated during CIK induction contained genes whose functions were associated with metabolism, circadian rhythm, and cytokine–cytokine receptor interaction (Fig. 5*A* and Table S2). KEGG pathways, including Toll-like receptor signaling, TNF signaling, cytosolic DNA sensing, and RIG-I–like receptor signaling pathways, intensively converged at a gene cluster that dramatically increased in response to interferon-γ priming and kept stable in the following culture (Fig. 5*B* and Table S3). For the genes transiently up-regulated by interferon-γ, the main functions of these genes included immune response and cell adhesion (Fig. 5*C* and Table S4). Notably, genes in T-cell receptor signaling and natural killer cell–mediated cytotoxicity were gradually increased in response to IL-2 and OKT3 (Fig. 5*D* and Table S5). Functions linked to cell cycle promotion and DNA replication were all induced between day 1 and day 7 (Fig. 5 (*E* and *H*) and Tables S6 and S9). Natural killer cell–mediated cytotoxicity is a pathway crucial to the function of CIK cells; genes involved in this pathway were both up- and down-regulated in the last 7 days of induction (Fig. 5 (*F* and *G*) and Tables S7 and S8).

### Sequential gene activation and repression in cell cycle and cytotoxicity

**Figure 6.**
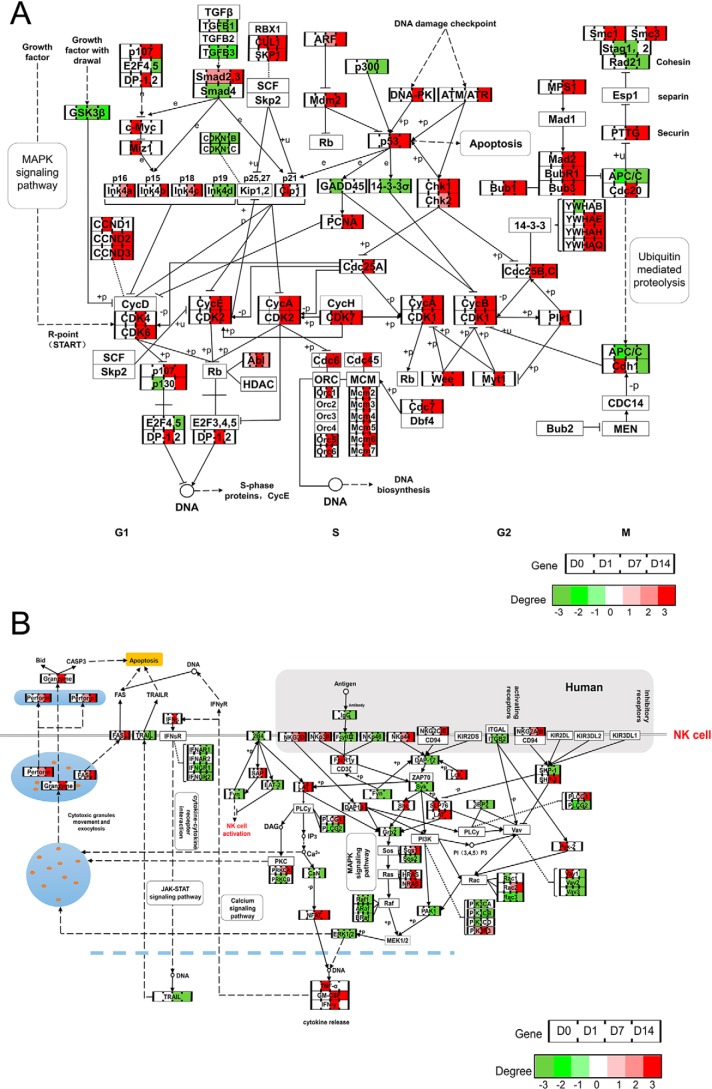
**The dynamic gene expression profiles of CIK cells involved in the cell cycle and the natural killer cell–mediated cytotoxicity pathway.** *A* and *B*, stage-specific expression of individual genes in pathway-orchestrated proliferation and antitumor function of CIK cells.

To probe the dynamic expression profiles and identify the hub genes controlling CIK functions, the time courses of gene expressions from PBMCs to CIK cells were aligned to the canonical cell cycle and natural killer cell–mediated cytotoxicity pathways (Fig. 6). Both TGFβ-1 and TGFβ-3 were dramatically down-regulated during CIK cell induction (Fig. 6*A*). Consistently, DNA replication–related genes, including *CDC45*, *ORC1*, *ORC6*, and *MCM2*, -*3*, -*4*, -*5*, and -*7*, specifically peaked at day 7, which indicated their roles in cell division. (Fig. 6*A*). Importantly, the expression profile map demonstrated that cyclins (D, E, A, and B) and cyclin-dependent kinase (1, 2, 4, 6, and 7) abruptly increased on days 7 and 14. Additionally, similar expression profiles were classified in genes that were involved in cell division, including *BUB1/3/R1*, *MAD2*, *SMC1/3*, *CDC25B/C*, *PTTG*, and *MPS1* (Fig. 6*A*). It was known that CIK cells resembled NK cells, which displayed their phenotypes and antitumor activity. Our data demonstrated that most of the activating receptors on NK cells, such as *NKG2D*, *NCR3*, *NCR2*, *NKG2A/B*, and *NKG2C/E*, gradually increased and peaked at day 14 (Fig. 6*B*). Importantly, the tumor cytotoxic molecules, including perforin, granzyme, and Fas ligand, were characteristically expressed in the late stages of CIK differentiation (Fig. 6*B*). By contrast, *TRAIL* (*TNFSF10*) was unexpectedly down-regulated at day 7, which implied that the tumor cytotoxicity of CIK cells was independent on TRAIL (Fig. 6*B*).

### Molecular atlas of tumor cytotoxicity and tumor promotion function during CIK induction

**Figure 7.**
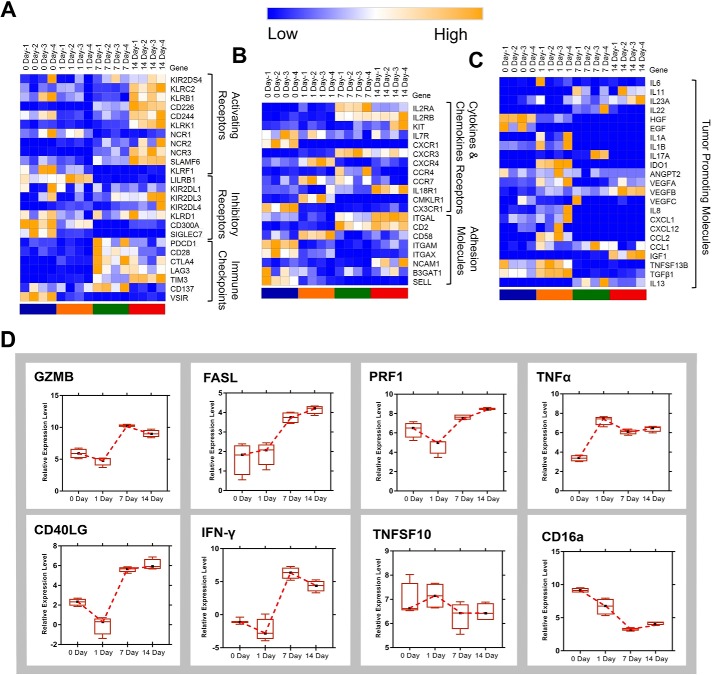
**Gene expression profiles across four time points in CIK cell production.** *A–C*, heat map of dynamic gene expression of activating receptors, inhibitory receptors, immune checkpoints, cytokine and chemokine receptors, adhesion molecules, and tumor-promoting molecules at days 0, 1, 7, and 14. *D*, relative expression level of tumor cytotoxic genes at each time point. *Error bars*, S.E.

To further investigate the antitumor activity and safety of CIK cells, time course–based expression analysis was performed among genes including receptors, immune checkpoint, adhesion molecules, and inflammatory factors. Besides *NKG2D* (*KLRK1*), activating receptors, such as *KIR2DS4*, *KLRC2*, *KLRB1*, *CD226*, *CD244*, *NCR2*, *NCR3*, and *SLAMF6*, were up-regulated in mature CIK cells (day 7) (Fig. 7*A*). Unlike other activating receptors, the expression of *NCR1* decreased in response to the stimulation of IL-2 and OKT3. In terms of inhibitory receptors, *LILRB1*, *KIR2DL1*, *KIR2DL4*, *KIR2DL1*, *CD300A*, and *SIGLEC7* were down-regulated in CIK cells compared with PBMCs (Fig. 7*A*). However, the expression of *KLRD1* (*CD94*) and *KIR2DL3* increased in CIK cells (Fig. 7*A*). Notably, we examined the expression profiles of the immune checkpoints during CIK cells differentiation. The results indicated that *PDCD-1* (*PD-1*), *CD28*, *CD137*, and *VSIR* were down-regulated in CIK cells, whereas *LAG3*, *CTLA4*, and *TIM3* were up-regulated (Fig. 7*A*). In addition, we found that the expression of *IL-2R*, *CXCR3*, and *IL-18R1* were increased, and other cytokines and chemokine receptors that were previously identified on NK cells were down-regulated in mature CIK cells (Fig. 7*B*). Adhesion molecules, including *ITGAL*, *CD2*, and *NCAM1*, were found to be highly expressed on CIK cells (Fig. 7*B*). To explore the tumor-promoting potential of CIK cells, we examined the expression of proinflammatory molecules that were reported to promote tumors. Fortunately, the results showed that the expressions of most tumor-promoting factors were depressed; however, *IL-23A*, *VEGFB*, and *IGF1* were up-regulated on day 14 (Fig. 7*C*). The expressions of antitumor molecules, including *GZMB*, *FASL*, *PRF1*, *TNF*α, *CD40L*, and *IFN-*γ, were increased in mature CIK cells (Fig. 7*D*).

### Selective validation of functional genes by flow cytometry

**Figure 8.**
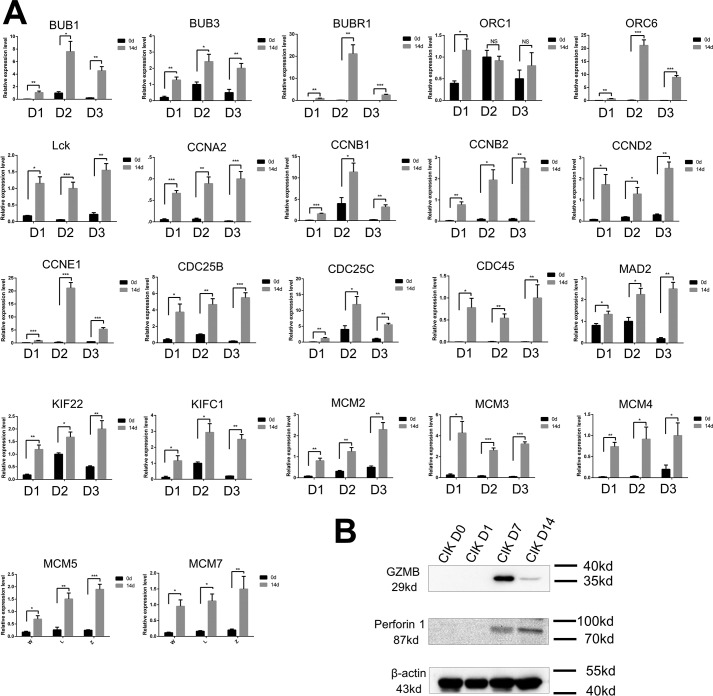
**qRT-PCR and Western blotting analysis of the expression of genes participating in cell proliferation, immune cell activation, and cytotoxicity.** *A*, qRT-PCR showed relative expression levels of genes involved in cell proliferation and CIK cell activation (*, *p* < 0.05; **, *p* < 0.01; ***, *p* < 0.001). *B*, Western blotting indicated the expression of GZMB and perforin 1 at days 0, 1, 7, and 14. *Error bars*, S.E.

**Figure 9.**
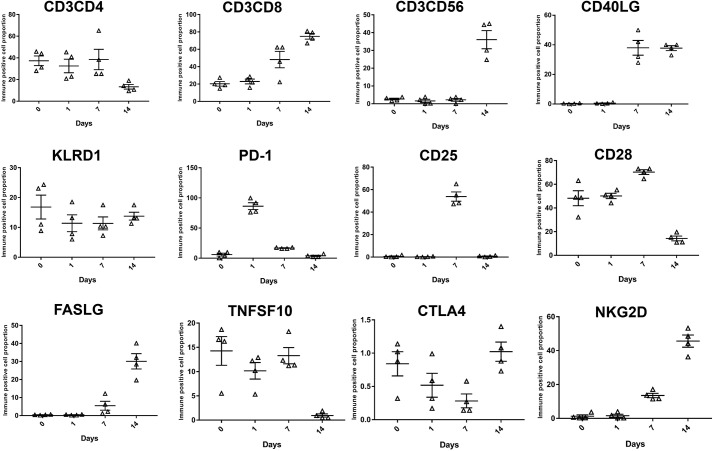
**Flow cytometry assay of selected markers of CIK cells identified by bioinformatic analysis.** *Error bars*, S.E.

To validate the gene expression, we selected several essential genes and examined their expressions by qRT-PCR, flow cytometry, and Western blotting. We analyzed the expression of 22 DGEs that participated in mitosis and T-cell activation. Except for *ORC6*, genes included in the qRT-PCR validation assay were significantly up-regulated in CIK cells obtained at day 14 compared with PBMCs (Fig. 8*A*). Moreover, we examined the expression of GZMB and PRF1 among CIK cell samples that were obtained at four time points. GZMB and *PRF1* were detectable at day 7 and day 14. Interestingly, the expression of GZMB in CIK cells at day 7 was significantly higher than at day 14. However, opposite expression trend was observed in *PRF1* (Fig. 8*B*). Furthermore, we analyzed expression dynamics of some crucial genes by grouping cells. The results showed that CD3^+^CD8^+^ cell subsets increased in response to IL-2 and OKT3 stimulation; however, the differentiation of CD3^+^CD56^+^ CIK cells began at late stage of the induction. Low expression of CD4 was identified in mature CIK cells. Up-regulation of the activating receptor NKG2D started at day 7, and the inhibitory receptor KLRD1 displayed stable expression throughout the induction. T-cell activation markers CD25 and CD28 showed high expression at day 7; nevertheless, they were dramatically decreased at day 14 (Fig. 8). Interestingly, PD-1 was significantly up-regulated in response to interferon-γ and restored at day 14. Unlike PD-1, CTLA gradually decreased in the first 7 days and was up-regulated on day 14. In terms of tumor cytototxic genes, *FASL* and *CD40L* were up-regulated by IL-2 and OKT3; however, TRAIL was down-regulated in mature CIK cells (Fig. 9). Subsequently, we evaluated the tumor cytotoxicity of CIK cells harvested at different stages. The results indicated that CIK cells obtained at day 7 showed significantly greater cytotoxicity against lung and stomach adenocarcinoma than cells from day 14 (Fig. S3).

## Discussion

In recent years, tumor immunotherapies have made remarkable progress and are revolutionizing cancer treatment strategies. There are dozens of immunotherapies against cancer, which can be classified into five types: monoclonal antibodies, immune-checkpoint inhibitors, cancer vaccines, adoptive cell transfer, and cytokines. CIK cells were first discovered by Schmidt-Wolf at Stanford in the 1990s and exhibited high proliferative potential and cytotoxicity against multifarious tumors (19). A number of clinical trials of immunotherapies based on CIK cells have been widely performed in China in the past decade (20). However, some of these studies were not qualified and were performed without standardized quality control. Moreover, the sale of CIK cell–based immunotherapies and exaggeration of their efficacies on some types of tumors have greatly impeded their progress toward clinical applications. Therefore, a great amount of challenging work must be done before they are put into clinical use. Importantly, molecular evidence of proliferation, tumor cytotoxicity, and safety should be elucidated. Recently, the secretome of CIK cells derived from tumor patients was elucidated (21). It was reported that several groups of interleukins and secreted proteins from CIK cells were identified based on their secretion quantity. However, analytic study to understand antitumor function, cell proliferation, and safety of CIK cells is indispensable. Herein, we have presented a dynamic transcriptomic atlas, which provides molecular evidence of the functions and safety of CIK cells. Our results showed that dramatic expression changes were observed among a great number of genes during CIK cell differentiation.

By employing WGCNA, we identified two groups of DGEs that were strongly associated with the functions of T-cell activation and cell proliferation. Specifically, *LCK* kinase and *HSPD1P1* were crucial genes for T-cell differentiation that were significantly up-regulated. Convincing evidence indicated that the cytotoxic function of CIK cells was non-MHC–restricted and resembled the antitumor function of NK cells. NK cells possessed Src family kinases, including Fyn, Lck, Yes, and Src (22). Studies have shown that the activity of Src family kinases is highly redundant for cytokine secretion and antibody-dependent cell-mediated cytotoxicity (ADCC) in NK cells (23, 24). Lck is a member of the Src kinase family that plays a key role in lymphocyte activation and differentiation (25). It was reported that Lck was constitutively activated in mature T cells (26). A strong physical association existed between Lck and CD4 and between Lck and CD8, which indicated that Lck was a crucial signal transduction mediator of T-cell activation (27). Interestingly, chemical inhibition of Src family kinases could efficiently block NK cell activation, and anti-CD8 antibody could inhibit CD16-mediated ADCC in FC receptor–bearing cytotoxic T cells (24, 28). Our results indicated that the expression of Lck kinase was abundant and increased 20-fold in response to the stimulus of OKT3 and IL-2. Among CIK cells, there were over 70% CD8^+^ and 35% CD56^+^ T cells. However, the expression of CD16 was significantly down-regulated in mature CIK cells compared with PBMCs. It seemed that the activation and tumor cytotoxicity of CIK cells relied on CD8 and Lck kinase, which resembled T cell–mediated ADCC. Moreover, we identified *CD86*, *IL-18*, *HSPD1P1*, and *TRAF6* as differentially expressed between PBMCs and CIK cells. However, these genes were expressed with low abundances, although significant changes were observed.

During CIK cell production, cell proliferation is one of most important biological events. The functions of DGEs in the brown module were mainly associated with mitosis and cell cycle. Obviously, several kinesin family members with a similar expression pattern were classified, including *KIF22*, *KIFC1*, *KIF11*, *KIF15*, and *KIF20B* (Fig. 4*A*). These proteins were involved in spindle formation and movements of chromosomes during mitosis (29). We also identify a group of protein serine/threonine kinases in the brown module, which were required for mitotic spindle assembly, chromosome alignment, segregation, and cytokinesis (30). The protein kinases included *BUB1B*, *NEK2*, *BUB1*, *CIT*, and *AURKB* (Fig. 4*A*).

By time-course series analysis, there was a cluster of genes (series 6) that were down-regulated in response to OKT3 and IL-2. Function analysis indicated that natural killer cell–mediated cytotoxicity was the most significant pathway among these genes. By tracing the genes, we found that the expression of some self-antigens decreased, including *MICA* and *HLA-C/E/B/G*, which implied that the immunogenicity of CIK cells was reduced (31, 32). Notably, the cytotoxic genes, including *TRAIL* (*TNFSF10*) and its receptor (*TNFSF10D*), were significantly down-regulated by stimulus of OKT3 and IL-2. Probably, the antitumor function of CIK cells was TRAIL-independent. For the series 7 gene cluster, a few genes were identified to participate in NK cell–mediated cytotoxicity whose expression increased at late stage of CIK induction. We found that most of these genes were NK cell receptors. Among these genes, *NKG2D*, *KLRC2*, and *KLRC3* were significantly up-regulated in CIK cells, which may mediate their antitumor effect. Either KLRC2 or KLRC3 could form a complex with CD94, performing an important role in immunosurveillance by binding to HLA-E (33, 34). Furthermore, the expressions of other NK cell–activating receptors increased in CIK cells, including *KLRB1*, *CD226*, *NCR3*, and *SLAMF-1*. We also identified CD2 and ITGAL as crucial adhesion molecule receptors that were highly expressed in CIK cells. CD2 and ITGAL could mediate cell adhesion between immune cells and other types of cells and trigger cytotoxic function against tumor (35, 36). For the inhibitory receptors, *KIR2DL4* was up-regulated in CIK cells; in contrast, the expressions of *CD300A*, *SIGLEC7*, and *LILRB1* dramatically decreased. It was shown that CD300a could bind to phosphatidylserine, which was expressed on both tumor cells and apoptotic cells, resulting in decreased cytotoxicity of NK cells (37). Blockage of the interaction between CD300a and phosphatidylserine could increase cytotoxicity of NK cells. SIGLEC7 is a sialic acid–binding receptor that modulates NK cell cytotoxicity (38). Some gangliosides on tumor cells could inhibit antitumor function of NK cells in a SIGLEC7-dependent manner (39). Therefore, decreased expression of *CD300A* and *SIGLEC7* can facilitate the tumor cytotoxicity of CIK cells.

Importantly, the transcriptome analyzes implicated the molecular evidences of the tumor cytotoxicity of CIK cells. A set of tumor cytotoxic genes were up-regulated in CIK cells, including *GZMB*, *FASL*, *PRF1*, *TNF*α, *CD40L*, and *IFN-*γ. In addition, immune checkpoints were recognized as the major determinants of immunotherapies against cancer. The expressions of *PD-1* (*PDCD1*), *CD28*, *CTLA-4*, *CD137*, and *VSIR* (*C10orf54*) were decreased in CIK cells, whereas *LAG3* and *TIM3* were significantly up-regulated. Consistent with our study, it was reported that blockage of LAG3 and TIM3 could remarkably enhance the killing ability against the targets (40, 41). For safety considerations, we extracted the dynamic expression profiles of the inflammatory genes that were reported to promote cancer (42). Our results showed that most of the tumor-promoting genes were expressed at a low level in CIK cells. Compared with PBMCs, some genes were significantly down-regulated by cytokines used for CIK preparation, such as *EGF*, *HGF*, and *TGF*-β. Interestingly, we found that *IGF1* and *VEGFB* were abruptly up-regulated in CIK cells, which were harvested at day 14. It is true that overexpression of IGF-1 in cancer or stroma cells promotes cell cycle progression and anti-apoptosis. However, new evidence indicates that endogenous IGF-1 promotes NK cell cytotoxicity (43). VEGFB is a member of the vascular endothelial growth factor family that mediated angiogenesis in cancer (44). The role of VEGFB in CIK cell function is still elusive.

In summary, the transcriptomic atlas of CIK cells presented a bioinformatics platform to demonstrate and visualize molecular features of CIK cell production. By WGCNA, we identified two gene modules that mediate immune cell activation and cell mitosis. The results suggested that the activation and cytotoxicity of CIK cells relied on CD8 and its partner Lck kinase. The time-course series analysis implicated relative low immunogenicity of CIK cells due to decreased expression of some self-antigens. In contrast, we identified several crucial activating receptors and auxiliary adhesion receptors expressed on CIK cells that may work as sensors for tumor cells. Interestingly, the cytotoxicity of CIK cells depended on PRF1, GZMB, FASL, CD40L, and some cytokines. TRAIL is an antitumor molecule. Fortunately, most of the immune-checkpoint molecules and inflammatory tumor-promoting factors were depressed in CIK cells, which suggested their efficacies and safety in clinical trials.

## Experimental procedures

### Preparation of CIK cells and flow cytometry

All antibodies for the CIK cell phenotype assay were purchased from BD Biosciences and Biolegend. Cytokines for CIK cell production, including OKT3, IFN-γ, and IL-2, were from Miltenyi Biotec and Peprotech. The Bioethics Committee of Yan'an Affiliated Hospital of Kunming Medical University reviewed the study protocols and approved the drawing of peripheral blood from healthy volunteers after written informed consent for the purposes of CIK cell preparation and transcriptome analysis. All procedures in this study were performed in compliance with the Helsinki Declaration and national laws. Written informed consent was given by all volunteers participating in this study. The standard protocol of CIK cell generation was described previously. Briefly, PBMCs were purified from peripheral blood by standard Ficoll separation and cultured in RPMI 1640 growth medium at a density of 1 × 10^6^ cells/ml. The RPMI 1640 growth medium for CIK cells contained 10% fetal bovine serum, 2% l-glutamine, and antibiotics. The production of CIK cells was initiated by adding 1000 units/ml IFN-γ on day 0 and then 100 ng/ml OKT3 and 500 units/ml IL-2 within the following 14 days of culture. The CIK cells were propagated every 5 days with RPMI 1640 growth medium supplemented with anti-CD3 antibody and IL-2. The CIK cells were expanded for 14 days. The expression of corresponding markers was monitored by flow cytometry at days 0, 1, 7, and 14. The cells were harvested by centrifugation at a speed of 2000 rpm. The cell pellets were suspended with blocking buffer. After washing with blocking buffer, the cells were stained with corresponding mAbs for 30 min at room temperature. After staining, the cells were washed twice before FACS analysis.

### RNA extraction and whole-transcriptome sequencing

Total RNA was extracted from each sample using TRIzol reagent (Life Technologies, Inc.) based on the protocol from manufacturer. The quality was assessed by the Agilent2200 TapeStation system (Agilent). The library of each RNA sample was prepared by using the Ion Total RNA-Seq Kit version 2 according to the protocol provided by manufacturer (Life Technologies). The method was described previously (45). Briefly, poly(A)-containing mRNA was isolated from 5 μg of total RNA with Dynabeads (Life Technologies). The mRNA was fragmented using RNase III and purified. The fragmented RNA was hybridized and ligated with an ion adaptor. The RNA fragments were reverse-transcribed and amplified to double-stranded cDNA. Then the amplified cDNA was purified by a magnetic bead–based method, and the molar concentration was determined for each cDNA library. Emulsion PCR was performed using the template of the cDNA library. The Template-Positive Ion PI^TM^ Ion Sphere^TM^ particles were enriched and loaded on the Ion PI^TM^ chip for sequencing.

### Raw read processing

All of the sequencing reads were collected, and reads ≥50 bp that passed filtering were used for mapping. Masplicing was employed to perform RNA-Seq data mapping (46). The core program of Masplicing is Bowtie, which can identify the exon–exon splicing immediately and accurately. We applied the DEseq to identify the DGEs for the CIK cells with the following criteria: *p* < 0.05, false discovery rate <0.05, and log -fold change >1 or <−1 (47).

### WGCNA and GO tree analysis

We used the R WGCNA package to build a weighted gene co-expression network that included 7740 nodes (DGEs) (48). The network was created based on the relationships between all pairs of DGEs by measuring the expression similarity. By WGCNA, we determined connectivity between two nodes by the similarity values. The weights ranged from 0 (strongly negatively correlated) to 1 (strongly positively correlated). To identify coexpression modules, the correlation matrix is transformed to an adjacency matrix. The interconnectedness for each pair of genes was determined by topological overlap measurement. Subsequently, we performed gene ontology analysis of the modules and picked modules that were functionally related with CIK cells. Then GO trees were built based on the significance of each GO term, the relationships among them, and the degree of interactions for each node (49).

### Series cluster analysis, pathway analysis

According to different expression change tendencies of genes at different time points, we have identified a set of unique model expression tendencies. The raw expression values were converted into log_2_ ratio. Using a strategy for clustering short time-series gene expression data, we could define some unique profiles. Pathway analysis was performed for each expression series cluster. Pathway analysis was used to determine the significant pathways that were associated with functions of CIK cells (50). We employed Fisher's exact test and the χ^2^ test to select the significant pathway, and the threshold of significance was defined by *p* value. The significant pathway was identified by *p* value < 0.05.

### Validation assay

The validation assay of crucial genes that may participate in cell proliferation, cell activation, and cytotoxicity was performed by qRT-PCR, Western blotting, and flow cytometry. The total RNA was extracted, and the first strand of cDNA was synthesized with adjusted concentration of RNA. The expression of corresponding genes was examined by qRT-PCR based on EVA Green Supermix (Bio-Rad). All of the primers were purchased from GeneCopoeia. Cell samples obtained from all time points were treated with cell lysis buffer, and the concentrations of total proteins were determined by a BCA-based method. For the immunoblotting of cytotoxic genes, the samples were analyzed by a 10% SDS-polyacrylamide gel loaded with equal amounts of protein. The proteins were electrotransferred to polyvinylidene difluoride membrane at 40 V for 100 min. Subsequently, the membrane was blocked with 5% skimmed milk in PBST overnight. Then the membranes were incubated with primary antibodies against GZMB (MAB3070, Merck Millipore), perforin 1 (sc-373943, Santa Cruz Biotechnology), and β-actin (ab6276, Abcam). The horseradish peroxidase–conjugated secondary antibodies were added after three-time PBST washing. After incubating, the membranes were washed thoroughly with PBST four times. Then the bands were visualized by an enhanced chemiluminescence kit (Merck Millipore). The expression dynamics of surface markers during CIK cell induction were analyzed by flow cytometry. Briefly, the cells were collected by centrifugation at a speed of 2000 rpm. The cell pellets were suspended with blocking buffer. After washing with blocking buffer, the cells were stained with corresponding mAbs for 30 min at room temperature. After staining, the cells were washed twice before FACS analysis.

## Author contributions

M. M. and J. T. conceptualization; M. M., L. L., R. L., J. T., and Z. H. data curation; M. M., L. L., J. S., W. T., and J. T. formal analysis; L. L., R. L., W. W., Y. C., Y. X., R. H., K. Z., W. H., Lili Yang, S. L., J. S., H. G., Y. Z., and Z. H. methodology; L. L., K. Z., Y. Z., and Li Yang writing-review and editing; R. L. and J. T. funding acquisition; R. L., R. H., H. G., and Li Yang writing-original draft; W. W., W. H., S. L., and Z. H. investigation; K. Z. validation; K. Z. visualization.

## Supplementary Material

Supporting Information

## Footnotes

4 The abbreviations used are:

CIK: cytokine-induced killer
TIL: tumor-infiltrating lymphocyte
NK: natural killer
CTL: cytotoxic T lymphocyte
PBMC: peripheral blood mononuclear cell
RPKM: reads per kilobases per million reads
DGE: differentially expressed gene
WGCNA: weighted correlation network analysis
GO: gene ontology
qRT-PCR: quantitative RT-PCR
ADCC: antibody-dependent cell-mediated cytotoxicity
EGF: epidermal growth factor
TGF: transforming growth factor
IFN: interferon.
