# Supplementary material for: A dynamic transcriptomic atlas of cytokine-induced killer cells
Source: J Biol Chem. 2018 Oct 17;293(51):19600–12. doi: 10.1074/jbc.RA118.003280 (PMC6314136; doi:10.1074/jbc.RA118.003280)
Supplement: Supporting Information [file supp_293_51_19600__index.html]

A dynamic transcriptomic atlas of cytokine-induced killer cells — Transcriptome analysis of cytokine induced killer cells — Supporting Information 

# A dynamic transcriptomic atlas of cytokine-induced killer cells

## Supporting Information

- table S1 - Relative expression level of CIK cells at different time points
- Table S2 - Pathway-Analysis\_Series\_1
- table S3 - Pathway-Analysis\_Series\_2
- table S4 - Pathway-Analysis\_Series\_3
- table S5 - Pathway-Analysis\_Series\_4
- table S6 - Pathway-Analysis\_Series\_5
- table S7 - Pathway-Analysis\_Series\_6
- table S8 - Pathway-Analysis\_Series\_7
- table S9 - Pathway-Analysis\_Series\_8
- Supplemental Figure 1
- Supplemental Figure2
- Supplemental Figure3
