# Supplementary figures and images for: A dynamic transcriptomic atlas of cytokine-induced killer cells
Source: J Biol Chem. 2018 Oct 17;293(51):19600–12. doi: 10.1074/jbc.RA118.003280 (PMC6314136; doi:10.1074/jbc.RA118.003280)

A

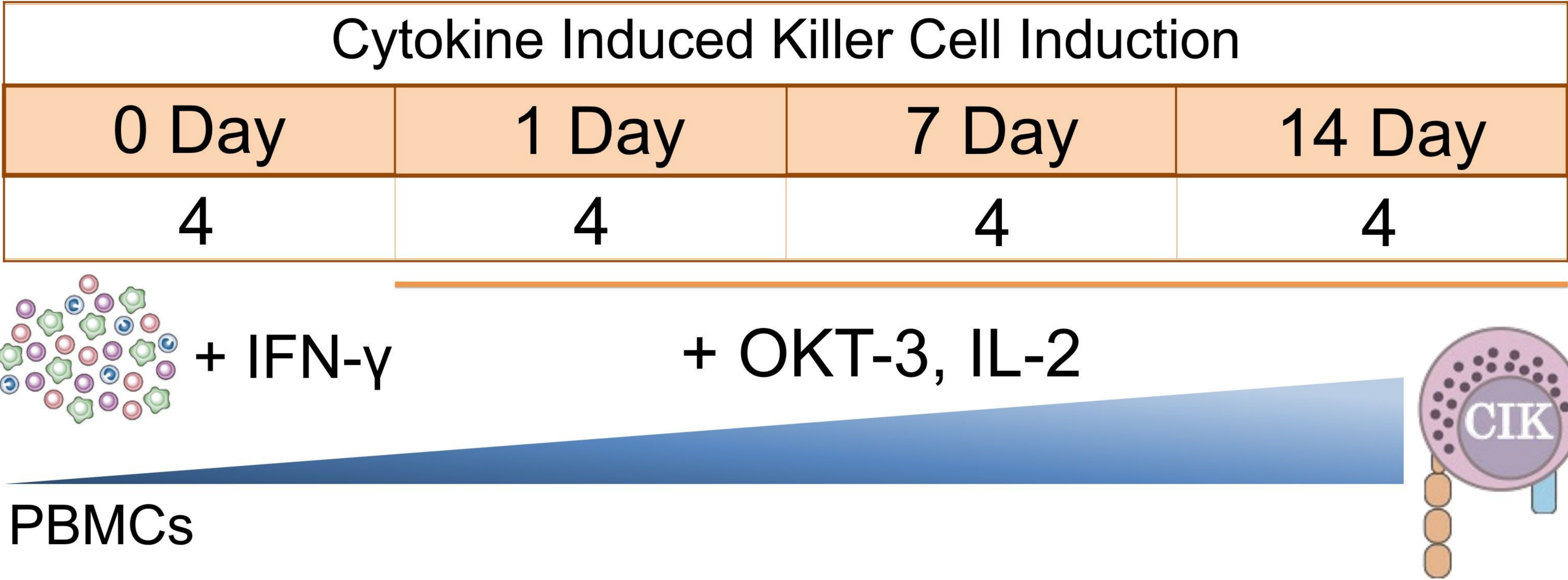

B

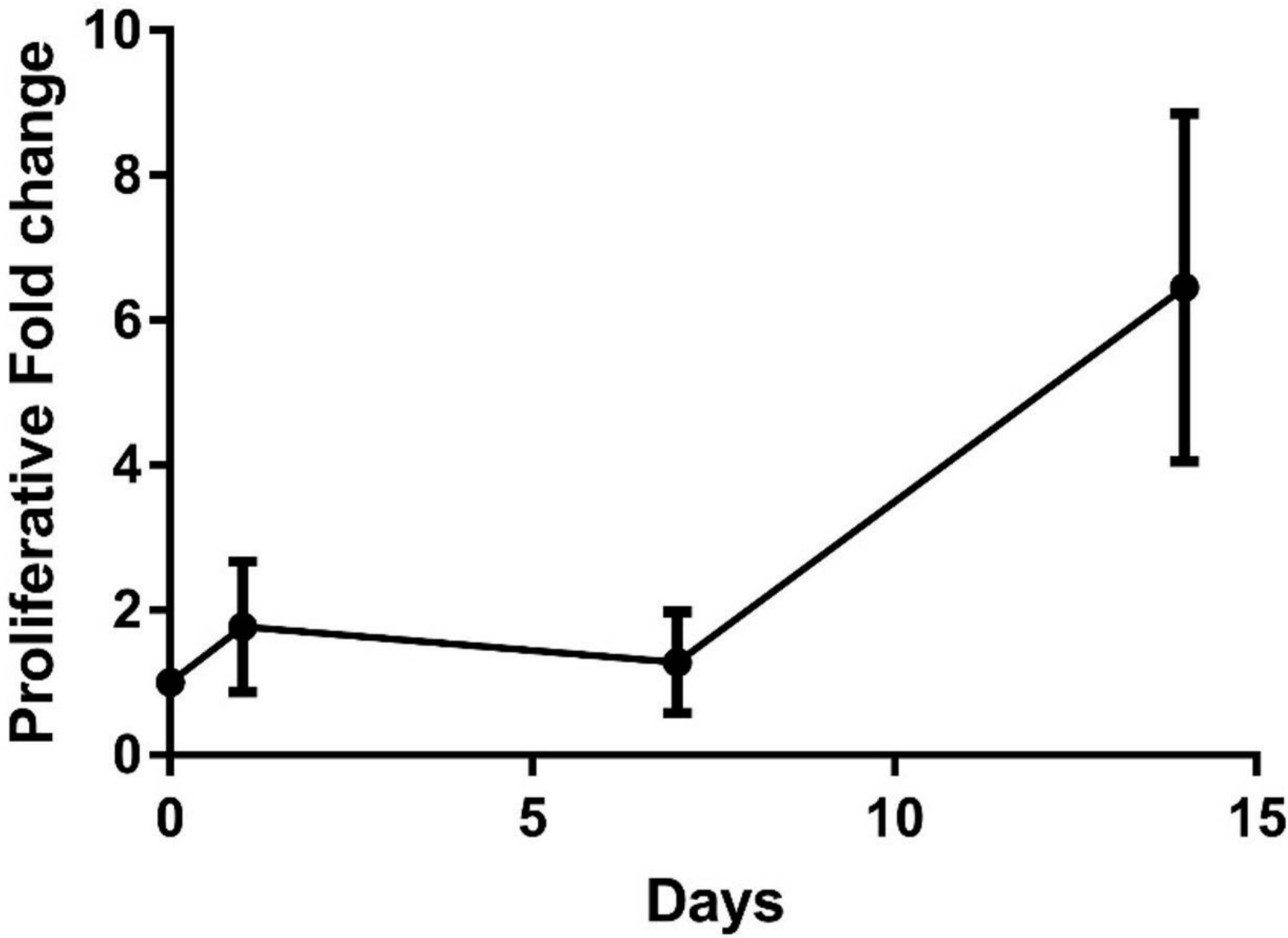

C

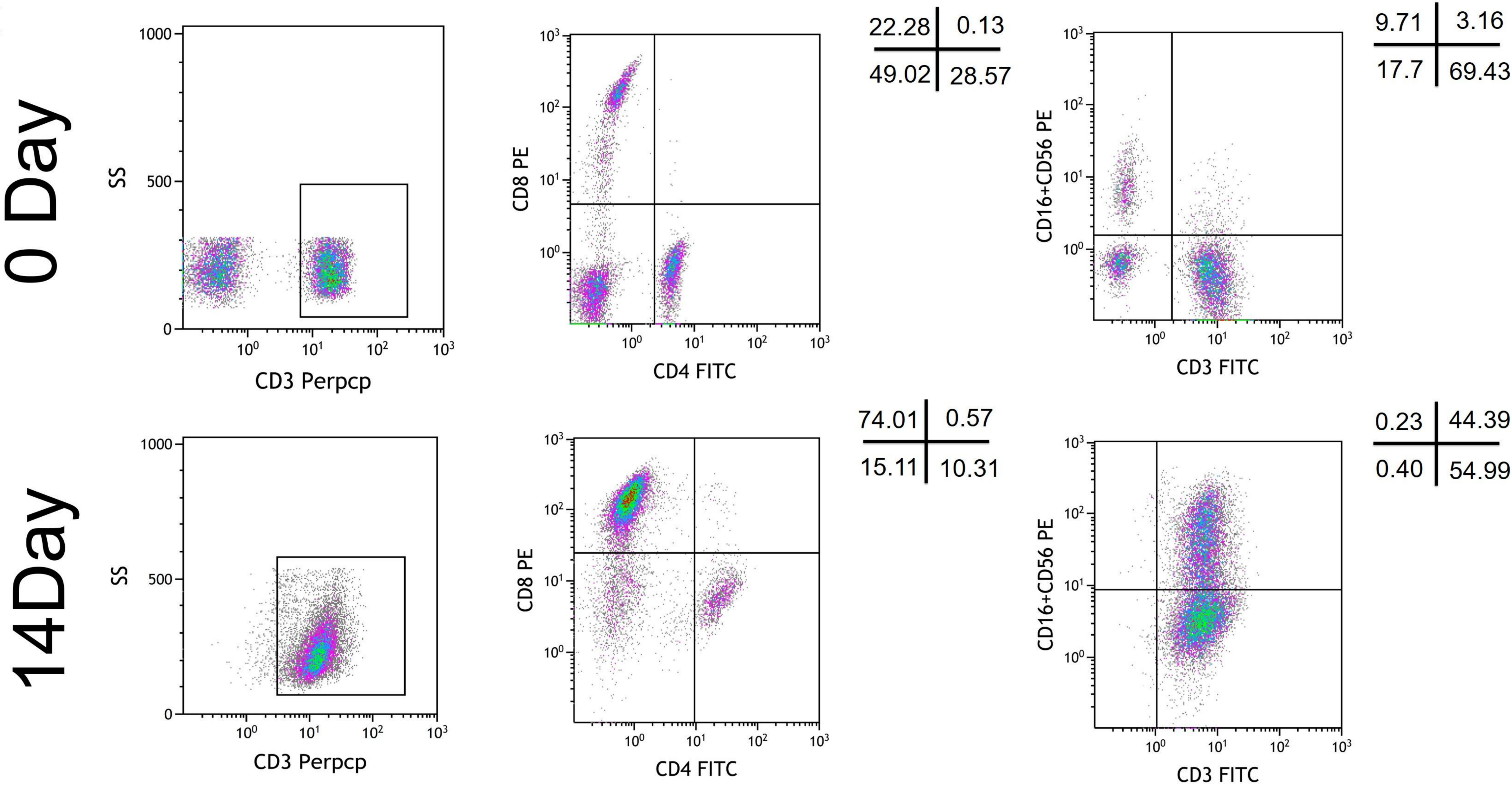

Supplement: Supporting Information [file supp_RA118.003280_Supplemental_Figure1.pdf]

**A**

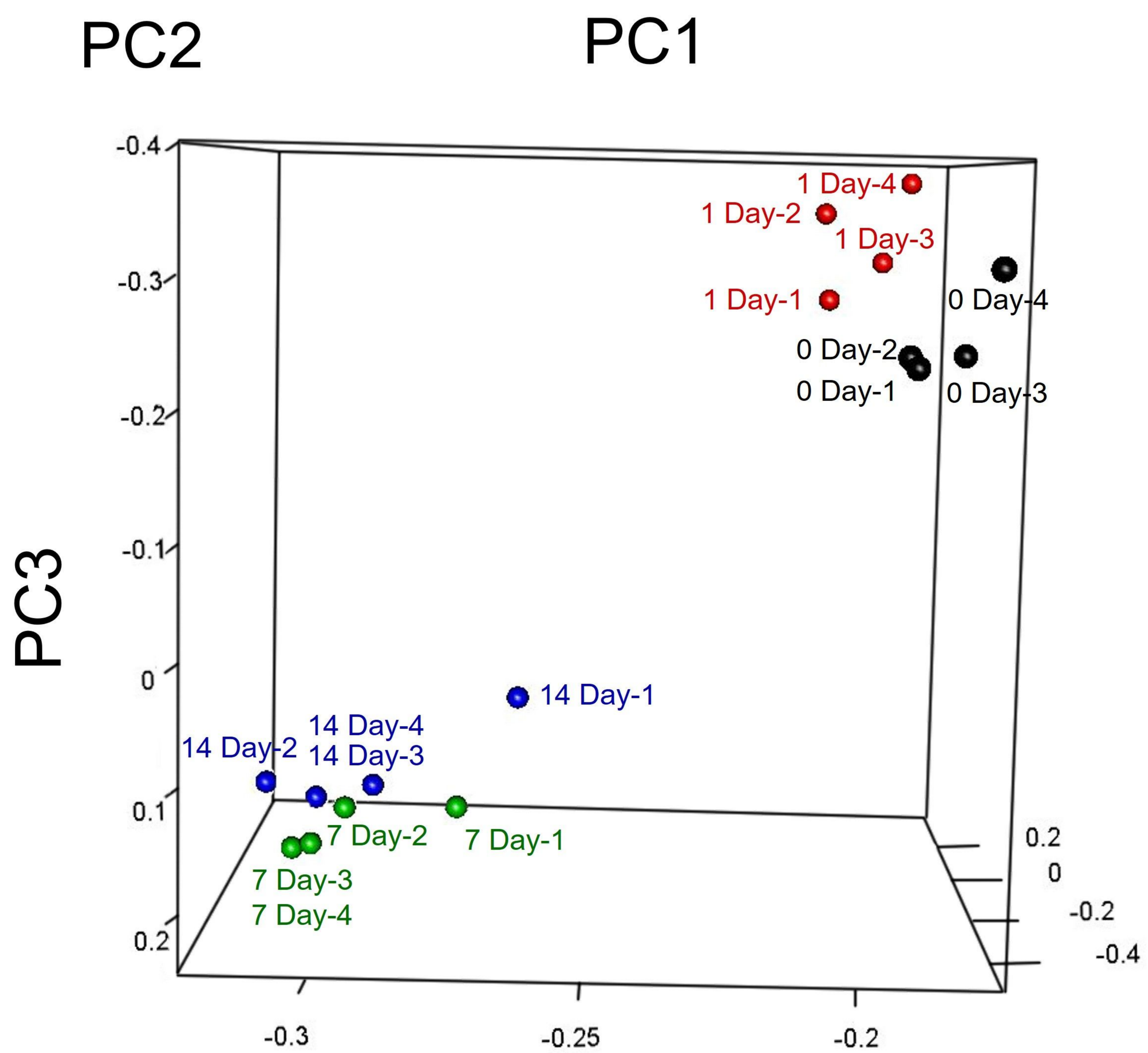

**B**

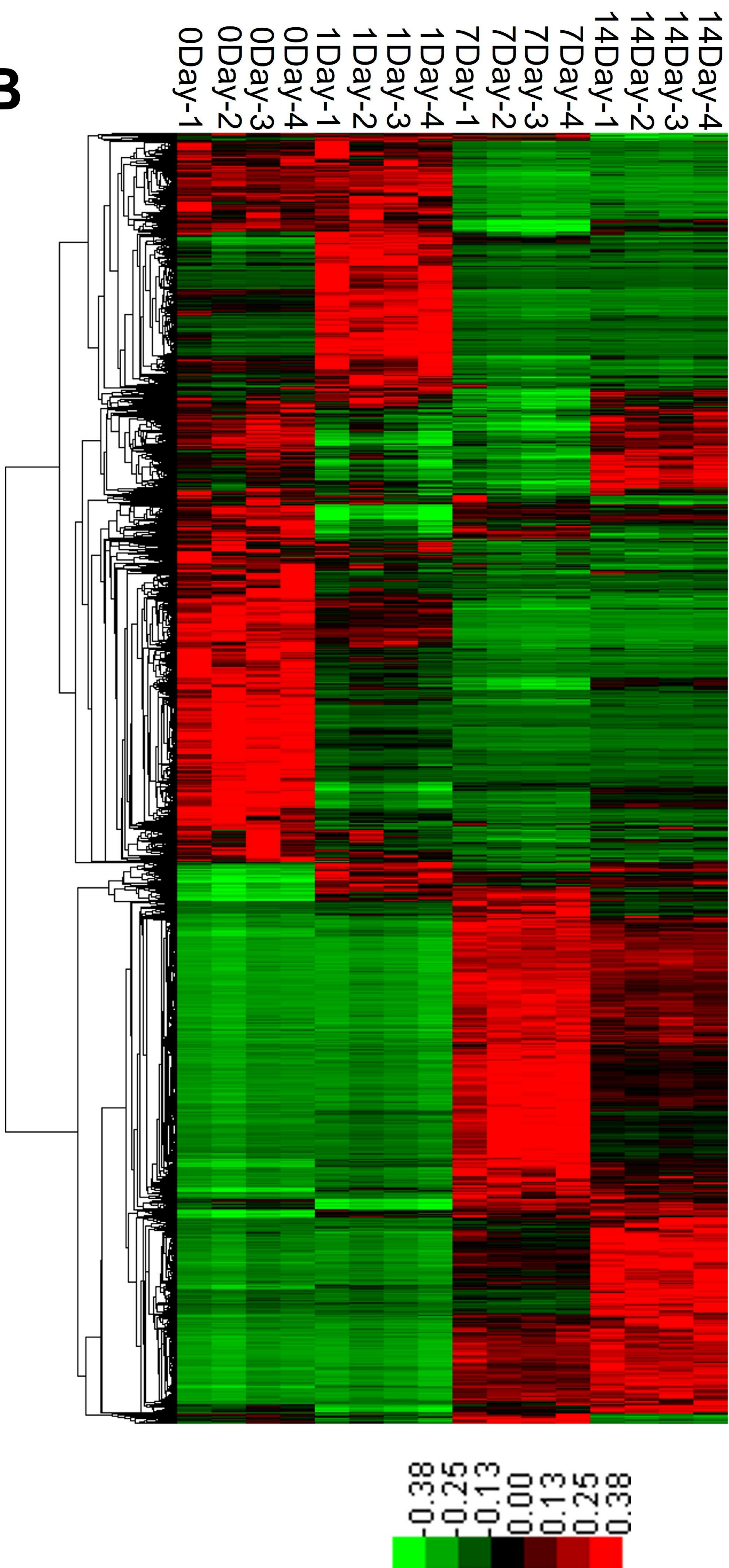

Supplement: Supporting Information [file supp_RA118.003280_Supplemental_Figure2.pdf]

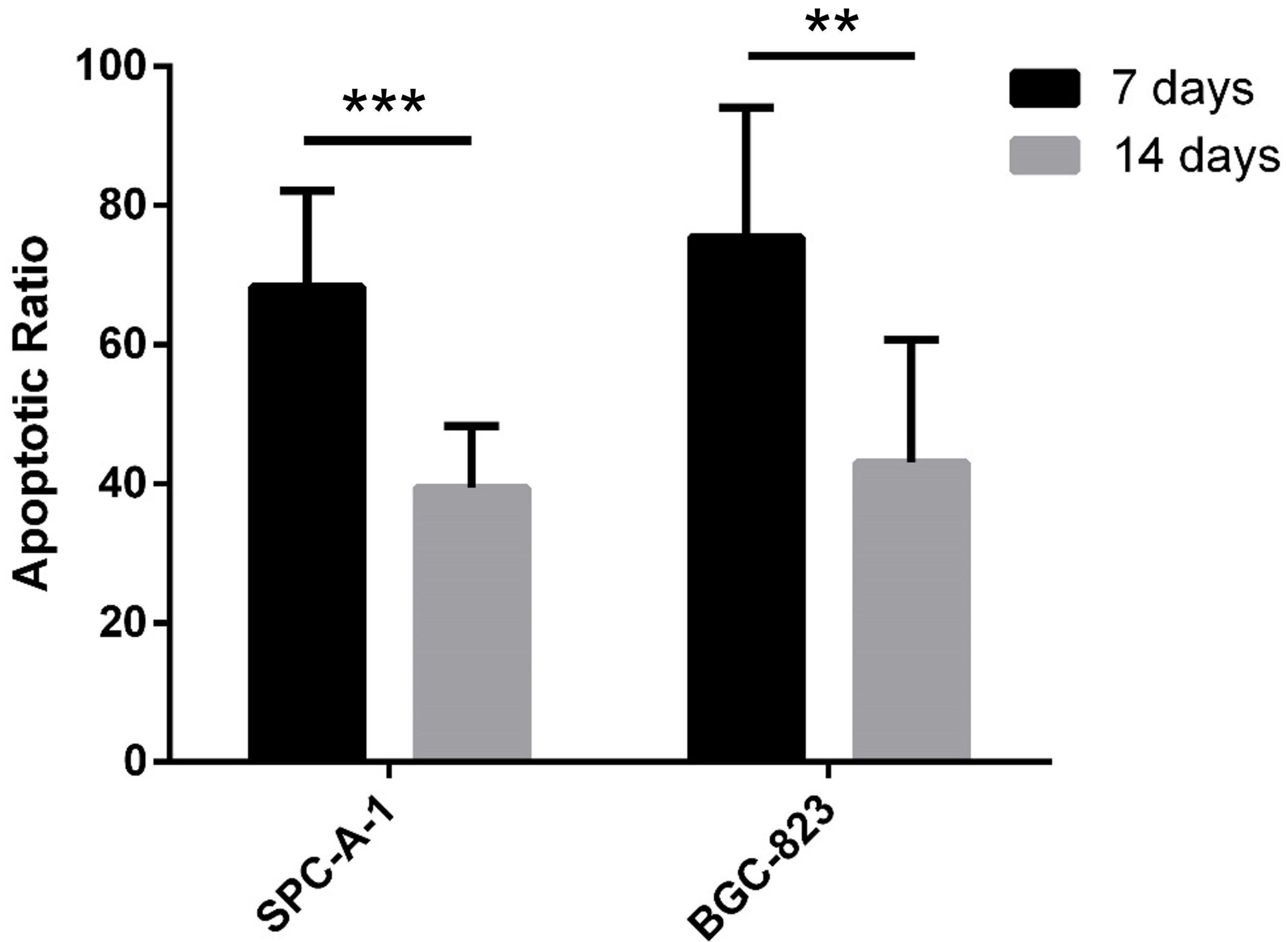

Supplement: Supporting Information [file supp_RA118.003280_Supplemental_Figure3.pdf]
